# Claudin-2 enhances human antibody-mediated complement-dependent cytotoxicity of porcine endothelial cells by modulating antibody binding and complement activation

**DOI:** 10.3389/fimmu.2025.1547512

**Published:** 2025-02-18

**Authors:** Weilong Li, Fang Yang, Dexin Yang, Zhuoheng Song, Zigan Xu, Jinmei Wu, Yanmei Li, Zixi Chen, Peishan Chen, Yeye Yu, Ting Xie, Cuishan Yang, Liying Zhou, Shaodong Luan, Hanchao Gao

**Affiliations:** ^1^ Department of Nephrology, Shenzhen Longhua District Central Hospital, Shenzhen Longhua District Key Laboratory for Diagnosis and Treatment of Chronic Kidney Disease, Shenzhen, Guangdong, China; ^2^ Department of Nephrology, The First Affiliated Hospital of Jinan University, Guangzhou, Guangdong, China; ^3^ Department of obstetrics, Shenzhen Longhua District Central Hospital, Shenzhen Longhua District Key Laboratory for diagnosis and treatment of chronic kidney disease, Shenzhen, Guangdong, China

**Keywords:** xenotransplantation, immune rejection, Claudin-2, PIECs, PAECs, complement, antibody, inflammation

## Abstract

**Background:**

Immune rejection represents a significant barrier to transplantation, especially in the context of xenotransplantation. Endothelial cells (ECs) derived from pigs serve as the initial barrier against the human immune system in xenotransplantation. Tight junction proteins are essential components of endothelial cell tight junctions; however, their role in xenotransplantation has been less thoroughly investigated. Claudin-2, a key tight junction protein, was investigated here for its role in human antibody-mediated complement-dependent cytotoxicity (CDC).

**Methods:**

Using an *in vitro* model of human antibody-mediated CDC, we assessed the effect of Claudin-2 on porcine aortic endothelial cells (PAECs) and porcine iliac endothelial cells (PIECs). Claudin-2 expression was either knocked down or overexpressed in these cells. A flow cytometry assay was used to evaluate C3c, C9, and the C5b-9 deposition, as well as the extent of human IgM and IgG binding to PIECs. The mRNA levels of complement regulators (CD46, CD55, CD59, Factor H, Factor I) were quantified by real-time PCR.

**Results:**

The loss of Claudin-2 protected PAECs and PIECs from human antibody-mediated CDC, while the overexpression of Claudin-2 enhanced the cytotoxicity in PAECs and PIECs within the same model. Unexpectedly, the loss or overexpression of Claudin-2 did not influence the mRNA expression levels of complement regulators (CD46, CD55, CD59, Factor H, and Factor I). Importantly, the loss of Claudin-2 significantly decreased the deposition of the C5b-9 complex, commonly referred to as the membrane attack complex (MAC), whereas the overexpression of Claudin-2 enhanced the deposition of the C5b-9 complex, indicating that Claudin-2 facilitates complement activation. Furthermore, the loss of Claudin-2 resulted in a decrease in the deposition of C3c and C9 on PIECs. Moreover, Claudin-2 enhanced human antibody binding to porcine ECs, as evidenced by increased IgG and IgM binding.

**Conclusion:**

Our findings indicate that Claudin-2 enhances the cytotoxicity of porcine ECs through modulating antibody binding and complement activation. The deficient of Claudin-2 in genetically modified pigs is likely to protect porcine ECs and enhance xenograft survival in pig-to-human organ or tissue xenotransplantation.

## Introduction

Organ transplantation is a crucial therapeutic option for patients with end-stage organ failure (1). However, the shortage of human donor organs is the primary limitation of organ transplantation. Xenotransplantation, the process of transplanting organs or tissues from one species to another, holds significant promise for addressing the shortage of human organ donors (2). Xenotransplantation involves the transplantation of organs, tissues, or cells from non-human animals, often pigs, into human recipients (3). Pigs are particularly appealing due to their physiological similarities to humans, rapid growth rates, and the ability to produce genetically modified organisms (4, 5). Despite its potential, immune rejection remains a formidable challenge, hindering the success of xenotransplantation (6–8). In pig-to-human xenotransplantation, porcine endothelial cells (ECs) represent the first line of defense against the human immune system (9, 10). Injury and dysfunction of ECs can lead to inflammation and coagulation, subsequently decreasing the survival time of xenograft in recipients.

The complement system represents a crucial component of the innate immune response, playing a significant role in host defense against pathogens (11). This complex network consists of over thirty proteins and are tightly regulated. Activation of the complement system occurs through three distinct pathways: classical, lectin, and alternative pathways, which converge on the central component C3. Each pathway initiates a cascade of proteolytic events, leading to the formation of key effector molecules (11, 12). Pig xenoantigens on the vascular ECs bind to human antibodies, activating the complement system and inducing antibody-mediated complement-dependent cytotoxicity (CDC) (13). When human antibodies activate the complement system, the resulting complement fragments and complexes transmit stimulatory signals, leading to the formation of the membrane attack complex (MAC), which is composed of the complement proteins C5b, C6, C7, C8, and C9 (C5b-9) (12). This intricate structure integrates into the ECs membrane, forming a pore that compromises the cell’s integrity and results in the lysis of ECs.

Claudin-2, a protein within the Claudin family, is crucial for the formation and function of tight junctions in epithelial and endothelial cells (14). Claudin-2 is distinguished by its unique to form channels that selectively permit the passage of small cations, including sodium ions, thereby contributing to the regulation of ion balance and homeostasis across various tissues. Dysregulation of Claudin-2 expression has been associated with several pathological conditions, including inflammatory bowel disease, in which altered permeability contributes to disease progression (15).

Various tight junction genes have distinct roles in human antibody-mediated CDC. Previously, we found that TNF-α promoted human antibody-mediated CDC through decreasing Occludin expression, and Zo-1 did not affect human antibody-mediated CDC of PIECs (16). IL-4 protected porcine ECs from human antibody-mediated CDC partially through increasing the expression of Claudin-5 (17). Interestingly, we found that knockdown of Claudin-2 in PIECs using lentivirus suppressed human antibody-mediated CDC. Nevertheless, the function and mechanism of Claudin-2 in human antibody-mediated complement-dependent cytotoxicity (CDC) remain unclear.

Here, we found that Claudin-2 enhanced human antibody-mediated CDC in both PIECs and PAECs. Although Claudin-2 didn’t affect the expression of complement regulators (CD46, CD55, CD59, Factor H, and Factor I), it facilitated the binding of human antibodies and the activation of the complement system. The development of Claudin-2-deficient pigs could be a promising strategy to reduce inflammatory responses in xenotransplantation.

## Materials and methods

### Reagents and cell lines culture

Fluorescein isothiocyanate (FITC)-conjugated goat anti-human IgM, IgG or isotype-matched antibodies were obtained from Invitrogen (Carlsbad, CA, USA). FITC-conjugated goat anti-mouse IgG antibody was purchased from Jackson ImmunoReasearch Laboratories (West Grove, PA, USA). FITC-conjugated anti-C3c antibody, anti-C9 antibody, anti-Claudin-2 antibody, and anti-C5b-9 antibody were purchased from Abcam (Shanghai, China). Anti-pig CD46 antibody was from Bio-Rad (Hercules, CA, USA). Anti-GAPDH antibody, and anti-Cleaved Caspase 3 antibody were obtained from Cell Signaling Technology (Boston, MA, USA). Recombinant human TNF-α were purchased from R&D Systems (Minneapolis, MN, USA). Cycloheximide (CHX) and LNP0023 was purchased form Targetmol (Shanghai, China). FITC-conjugated 40 kDa dextran was from Sigma (Shanghai, China). Cell Counting Kit-8 (CCK8) was purchased from Dojindo Laboratories (Kumamoto, Japan).

Porcine iliac endothelial cells (PIECs) were obtained from the Type Culture Collection of the Chinese Academy of Sciences, and the cells were cultured with RPMI-1640 containing 10% (vol/vol) FBS, 1% (vol/vol) P/S at 37°C with 5% CO_2._ Porcine aortic endothelial cells (PAECs) were isolated from one Chinese Wuzhishan wild-type pig, and the cells were cultured as previously described (18).

### Human antibody-mediated complement-dependent cytotoxicity

The procedure of human antibody-mediated complement-dependent cytotoxicity has been previously reported (19). In brief, PIECs or PAECs (8×10^3^) were seeded into 96 well plates. After 48 hours, the supernatant was removed and replaced with RPMI-1640, containing 20% pooled human serum (experimental group), which were from several healthy volunteers (n=22, including all ABO blood types), or 20% heat-inactivated human serum (control group) for 2 hours, and then the viability of PIECs or PAECs was assessed with CCK8. The supernatant was removed and replaced with RPMI-1640 containing 10% CCK8. After 2 hours, the absorbance values of wells were measured at OD 450 using a multiscan GO spectrophotometer (Thermo Fisher). The percentage of cytotoxicity was calculated by the following formula:


$$\%\text{cytotoxicity }=\;\left(\text{OD}\;\text{of}\;\text{control}\;\text{group}-\text{OD}\;\text{of}\;\text{experimental}\;\text{group}\right)/\text{OD}\;\text{of}\;\text{control}\;\text{group}\times 100.$$

**Figure 1:**
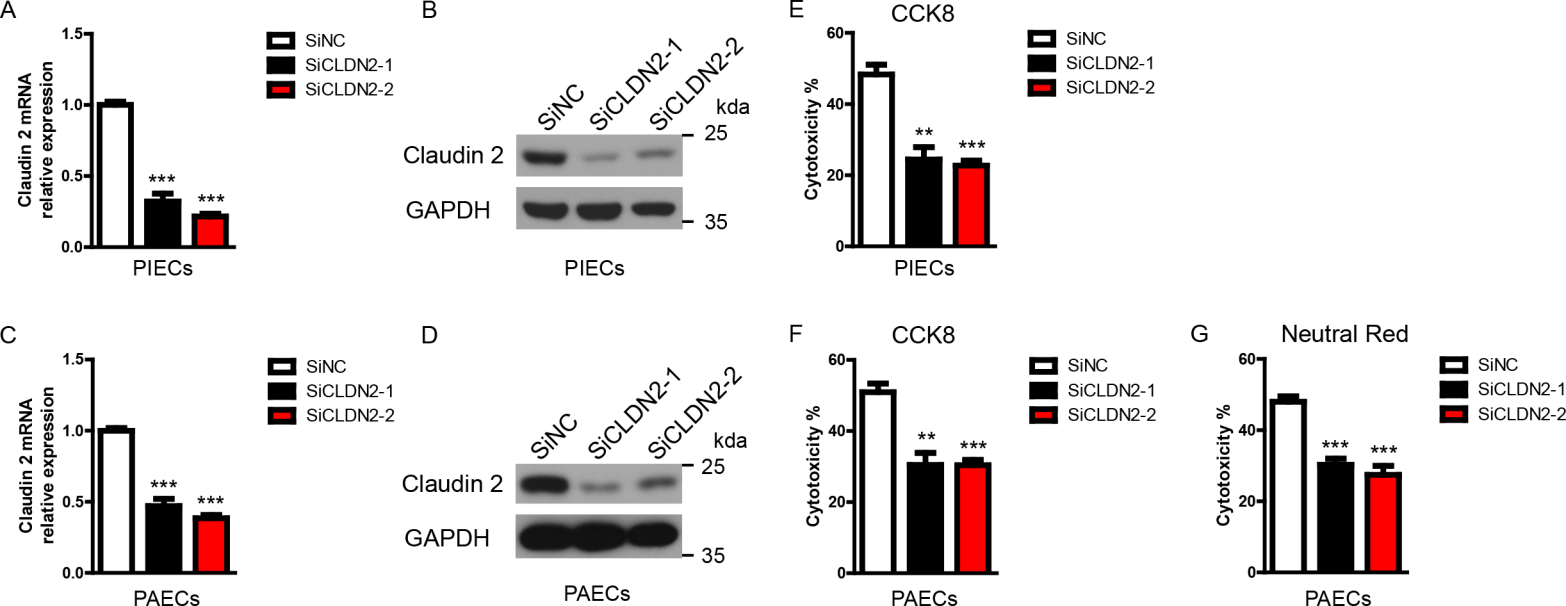
Loss of Claudin-2 suppresses the cytotoxicity of porcine ECs in a human antibody-mediated CDC model. **(A-D)** The siClaudin-2 oligos and control siRNA oligo were transfected to PIECs or PAECs. After 48h, total RNA of the transfected PIECs **(A)** or PAECs **(C)** was collected and the mRNA level of Claudin-2 was measured by RT-PCR. The lysates of the transfected PIECs **(B)** or PAECs **(D)** were analyzed by immunoblotting with antibodies against Claduin 2 and GAPDH. **(E)** PIECs were treated as in **(A)** and subsequently exposed to human serum to induce antibody-mediated CDC. The extent of cytotoxicity was assessed by CCK8. **(F, G)** PAECs were treated as in **(C)** and subsequently exposed to human serum to induce antibody-mediated CDC. The extent of cytotoxicity was assessed by CCK8 **(F)** or neutral red **(G)**. Data are representative of at least three independent experiments (mean ± SEM). **p < 0.01, ***p < 0.001 by Student’s t test.

Cytotoxicity was also assessed by neutral red staining (**Figure 1G**). The percentage of cytotoxicity was calculated using the same formula applied for CCK8.

### Loss of Claudin-2 in PIECs and PAECs

The scrambled control siRNA sequence is 5’-ggatccttgacaataccaa-3’. The two sequences for porcine Claudin-2 gene knockdown are 5’-gcatcatttcctccctgtt-3’ and 5’-tctcctcgttggcctgtat-3’. The siClaudin-2 oligonucleotides and control oligonucleotides were transfected into PIECs or PAECs using jetPrime^®^ DNA and siRNA Transfection Reagent (Polyplus transfection, Illkirch, France) following the manufacturer’s instructions. Forty-eight hours later, the transfected cells were utilized for experiments.

The sequences of siRNA double-stranded oligonucleotides were cloned into pLKO.1 lentiviral vector. The lentiviral and packaging vectors were transfected into HEK 293FT cells. Two days later, the virus was collected to infect PIECs and obtained ShNC, ShCLDN2-1, and ShCLDN2-2 cells. After four days, the infected cells were utilized for experiments.

### Adenoviral-mediated porcine Claudin-2 expression in PIECs and PAECs

An adenoviral mediated porcine Claudin-2 over-expression plasmid was constructed according to protocol. After the plasmid of adenovirus (Adv)-Claudin-2 was constructed, the recombinant plasmid Adv-Claudin-2, or empty vector (Adv-EV) was transfected into 293A cells for adenovirus packaging. The recombinant adenoviruses were amplified, purified, titrated, and diluted. The final concentration of adenovirus particles was diluted to 10^10^–10^11^ per ml of PBS. PIECs or PAECs were infected with Adv-Claudin-2, or Adv-EV for 72h, and then the infected cells were exposed to human serum to induce antibody-mediated CDC.

### Real time-PCR

Real-time PCR has been reported previously (20, 21). Total RNA of PIECs or PAECs was extracted with TRIzol^®^ Reagent (Invitrogen). The cDNA samples were synthesized with Transcript First-Strand cDNA Synthesis SuperMix (TransGene Biotech, Beijing, China). The levels of the interested genes were measured using TB Green^®^ Premix Ex Taq™ (Tli RNaseH Plus) (Takara Bio, Dalian, Liaoning, China). The expression levels of the genes were calculated by the 2^-ΔΔCt^ method and normalized to pig glyceraldehyde-3-phosphate dehydrogenase (GAPDH). Amplification of cDNA was performed on a 7500 Real-Time PCR system (Applied Biosystems, Foster City, CA, USA). The sequences of oligonucleotide primers were shown in **Supplementary Table S1**.

### Flow cytometry analysis

For CD46 expression, lentivirus or adenovirus infected PIECs (1×10^6^) were incubated with anti-CD46 antibody (1:100, v/v) for 1 hour at 4°C, with secondary antibody only as control. After washing with PBS, the cells were incubated with FITC-conjugated goat anti-mouse IgG (1:100, v/v) for 30 min at 4°C in the dark. After washing twice with PBS, the cells were resuspended in PBS containing 1% BSA and analyzed by BD FACS II flow cytometry. The gating strategy was illustrated in **Supplementary Figure S1**. The degree of CD46 expression was assessed by geometric mean fluorescence intensity (Gmean).

Human IgM and IgG binding assays were assessed using flow cytometry as previously described (22, 23). In brief, human serum from several healthy volunteers (n=22, including all ABO blood types) were mixed and heat-inactivated at 56°C for 30 min. Lentivirus or adenovirus infected PIECs (1×10^6^) were incubated with 20% heat-inactivated serum for 30 min at 37°C. After washing, 10% goat serum was applied to prevent non-specific binding. Subsequently, the cells were incubated with FITC-conjugated goat anti-human IgM or IgG antibodies (1:100, v/v) for 30 min at 4°C in the dark, with secondary antibodies only, without serum, serving as control group. The cells were subsequently treated and analyzed as described above. The gating strategy was similar to that of CD46 as described above. The degree of IgM and IgG binding to PIECs was quantified by Gmean.

For C9 and C5b-9 deposition, lentivirus or adenovirus infected PIECs (1×10^6^) were incubated with 20% human serum for 30 min at 37°C. Subsequently, the cells were incubated with anti-C9 antibody (1:100, v/v), or anti-C5b-9 (1:100, v/v) antibody for 1 hour at 4°C, with secondary antibody only as control. After washing with PBS, the cells were incubated with FITC-conjugated goat anti-mouse IgG (1:100, v/v) for 30 min at 4°C in the dark. The cells were subsequently treated and analyzed as described above. The gating strategy of C5b-9 was similar to that of CD46 as described above. The gating strategy of C9 was illustrated in **Supplementary Figure S2**.

For C3c deposition, lentivirus infected PIECs (1×10^6^) were incubated with 20% human serum for 30 min at 37°C. Subsequently, the cells were incubated with FITC-conjugated anti-C3c antibody (1:100, v/v) for 1 hour at 4°C in the dark, with secondary antibody only as control. The cells were subsequently treated and analyzed as described above. The gating strategy for C3c was shown in **Supplementary Figure S2**.

### Immunoblot analysis

The procedure of immunoblot analysis has been previously reported (24). In brief, PIECs were harvested and lysed for 30 min in ice-cold RIPA lysis buffer containing 10mM sodium fluoride (NaF), 1mM Na_3_VO_4,_ 1mM phenylmethylsulfonyl fluoride, and protease inhibitor cocktail (Roche, Indianapolis, IN, USA), and then separated by 10% SDS-PAGE. After the proteins were transfer onto polyvinylidene fluoride (PVDF) membranes (Millipore, Billerica, MA, USA), the PVDF membranes were then blocked at room temperature for 1 hour and subsequently incubated with primary antibody overnight at 4°C. After incubation with the secondary antibody for 1 hour at room temperature, the blots were visualized using enhanced chemiluminescence (ECL) detection reagents (Millipore).

### Permeability assay

Lentivirus infected PIECs were cultured on 3 μm pore Transwell filters until they reached confluence. Following two washes with PBS, 100 μg/ml of FITC-conjugated 40 kDa dextran was introduced into the apical compartment for 30 min. The fluorescence in the basolateral medium was quantified using a spectrofluorometer (Thermo Fisher) at an excitation wavelength of 492 nm and an emission wavelength of 520 nm.

### Statistical analysis

Experimental data are presented as the mean ± SEM. Statistical significance between the groups was calculated by using a two-tailed Student’s t test. *p* values <0.05 were considered significant.

## Results

### Loss of Claudin-2 protects porcine ECs from human antibody-medicated CDC

Previously, we found that knockdown of Claudin-2 in PIECs significantly decreased human antibody-mediated CDC (16). To further confirm the role of Claudin-2 in human antibody-mediated CDC model, we transfected Claudin-2 specific siRNA oligos to knockdown Claudin-2 expression in PIECs and PAECs, and then exposed to human serum (heat-inactivated serum as a control) to induce human antibody mediated CDC. We found that the mRNA and protein levels of Claudin-2 were significantly reduced in PIECs and PAECs (**Figures 1A–D**). Loss of Claudin-2 obviously reduced human antibody-medicated CDC in PIECs and PAECs assessed by CCK8 (**Figures 1E, F**). To confirm the results, neutral red was also used to assess the cytotoxicity in PAECs, and we obtained the similar results as CCK8 (**Figure 1G**).

### Porcine ECs infected with Adv-Claudin-2 promotes the cytotoxicity in a human antibody-mediated CDC model

**Figure 2:**
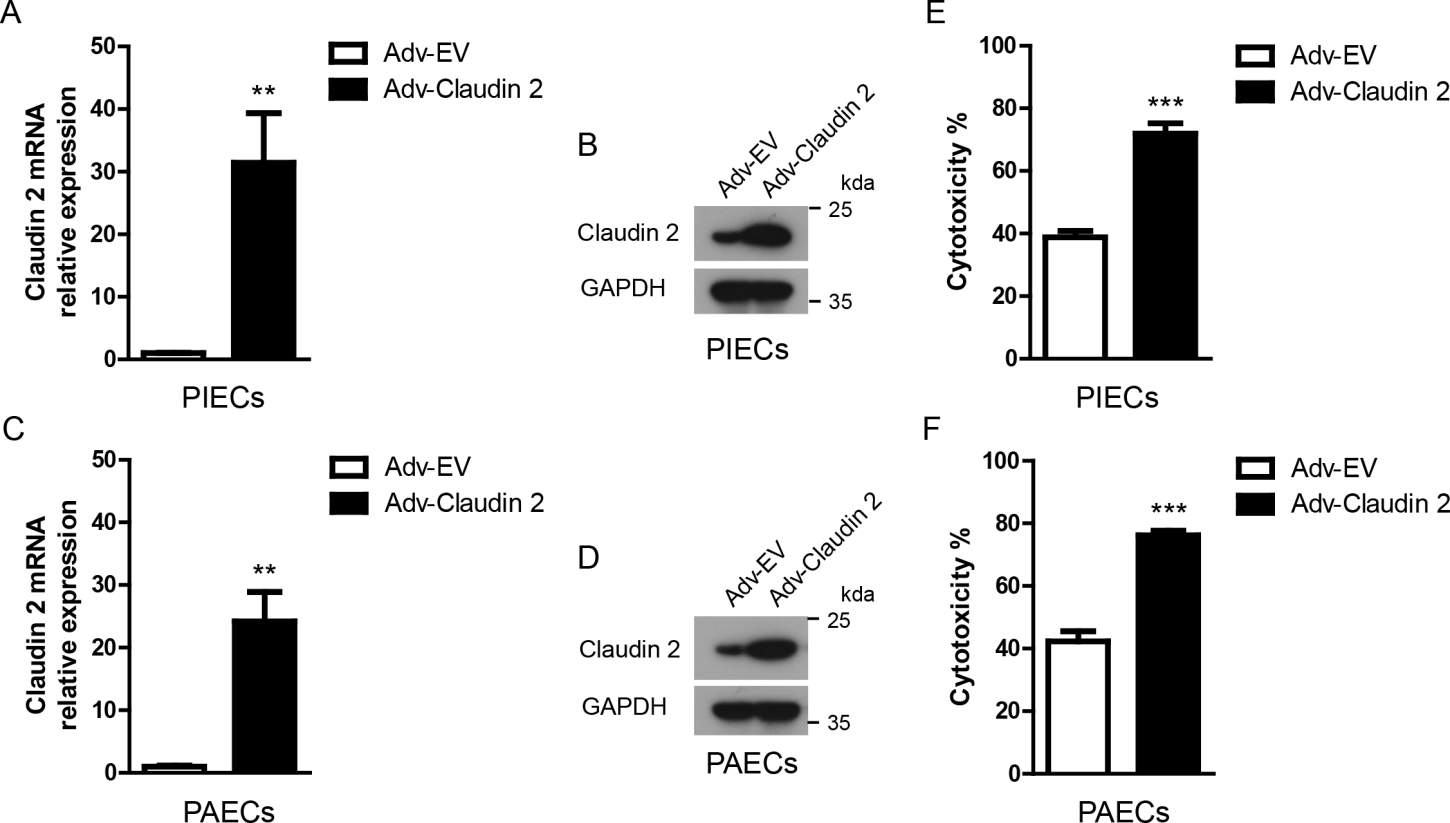
Claudin-2 ectopically expressed in porcine ECs using an adenoviral system protected ECs from human antibody-mediated CDC. **(A, B)** Claudin-2 was constructed using an adenovirus system. The adenovirus was packaged. PIECs were infected with Adv-EV (empty vector), or Adv-Claudin-2 for 72h, the total RNA was collected for RT-PCR analysis **(A)**. The lysates of the transfected PIECs were analyzed by immunoblotting with antibodies against Claduin-2 and GAPDH **(B)**. **(C, D)** PAECs were infected with Adv-EV, or Adv-Claudin-2 for 72h, the total RNA was collected for RT-PCR analysis **(C)**. The lysates of the transfected PAECs were analyzed by immunoblotting with antibodies against Claduin-2 and GAPDH **(D)**. **(E)** PIECs were treated as in A and subsequently exposed to human serum to induce antibody-mediated CDC. The extent of cytotoxicity was assessed by CCK8. **(F)** PAECs were treated as in C and subsequently exposed to human serum to induce antibody-mediated CDC. The extent of cytotoxicity was assessed by CCK8. Data are representative of at least three independent experiments (mean ± SEM). **p < 0.01, ***p < 0.001 by Student’s t test.

We overexpressed porcine Claudin-2 in PIECs and PAECs with an adenoviral system. The mRNA and protein expression levels of Claudin-2 were largely upregulated in PIECs or PAECs infected with Adv-Claudin-2 (**Figures 2A–D**). Adenovirus mediated-Claudin-2 expression in PIECs and PAECs significantly increased the cytotoxicity in the human antibody-mediated CDC model (**Figures 2E, F**). These data suggest that Claudin-2 promotes human antibody-mediated CDC of porcine ECs.

### Claudin-2 does not modulate the expression of complement regulators

**Figure 3:**
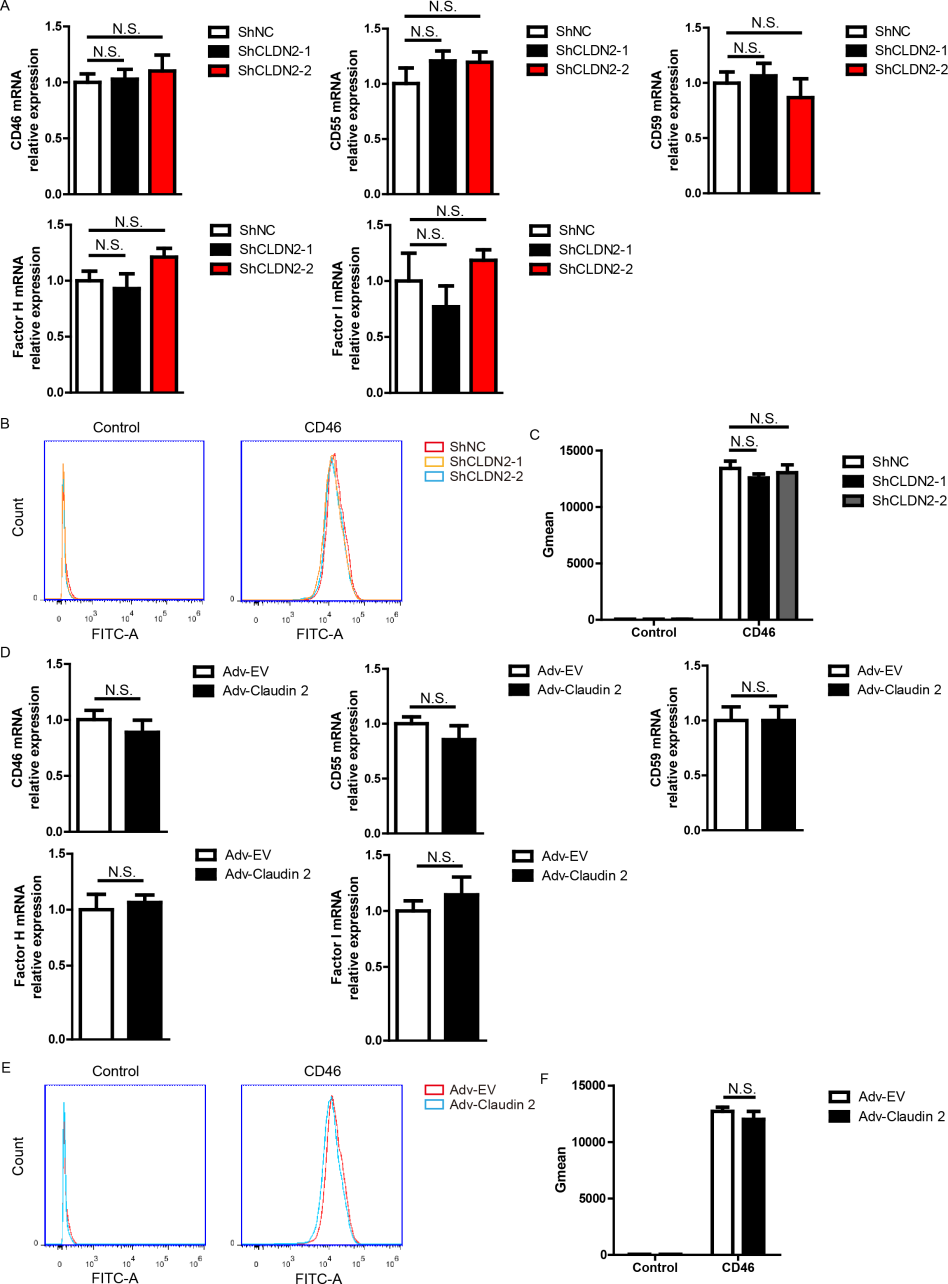
In PIECs, Claudin-2 did not affect the expression of CD46, CD55, CD59, Factor H, or Factor I. **(A)** PIECs were infected with control lentivirus (ShNC) or with lentivirus expressing Claudin-2 specific siRNA(ShCLDN2-1, or ShCLDN2-2). After 4 days, total RNA was collected and the mRNA level of CD46, CD55, CD59, Factor H, or Factor I was analyzed by RT-PCR. **(B, C)** PIECs were treated as in A and analyzed with flow cytometry to assess the protein level of CD46 **(B)**. The quantitation data were presented by geometric mean fluorescence intensity (Gmean) **(C)**. **(D)** PIECs were infected with Adv-EV, or Adv-Claudin-2 for 72h, the total RNA was collected and the mRNA level of CD46, CD55, CD59, Factor H, or Factor I was analyzed by RT-PCR. **(E, F)** PIECs were treated as in D and analyzed with flow cytometry to assess the protein level of CD46 **(E)**. The quantitation data were presented by Gmean **(F)**. Data are representative of at least three independent experiments (mean ± SEM). N.S. means no significance.

It is well-established that complement regulators, such as CD46, CD55, CD59, Factor H, Factor I, and CD35, suppress complement activation, thereby reducing human antibody-mediated CDC (25). We asked whether Claudin-2 promoted the cytotoxicity in the human antibody-mediated CDC model through modulating the expression of complement regulators. And we found that loss of Claudin-2 did not affect the mRNA levels of CD46, CD55, CD59, Factor H, and Factor I in PIECs (**Figure 3A**). Owing to its low abundance, the detection of CD35 mRNA in PIECs was not achieved by RT-PCR (data not shown). We also found that loss of Claudin 2 did not influence the protein level of CD46 in PIECs (**Figures 3B, C**). Consistently, PIECs infected with Adv-Claudin-2 also did not alter the mRNA levels of CD46, CD55, CD59, Factor H, and Factor I in PIECs (**Figure 3D**). Additionally, overexpression of Claudin 2 did not influence the protein level of CD46 in PIECs (**Figures 3E, F**). The data demonstrates that Claudin-2 does not modulate the expression of complement regulators, and complement regulators were not a major factor causing Claudin-2 to promote the killing of porcine ECs.

### Claudin-2 promotes membrane attack complex formation

**Figure 4:**
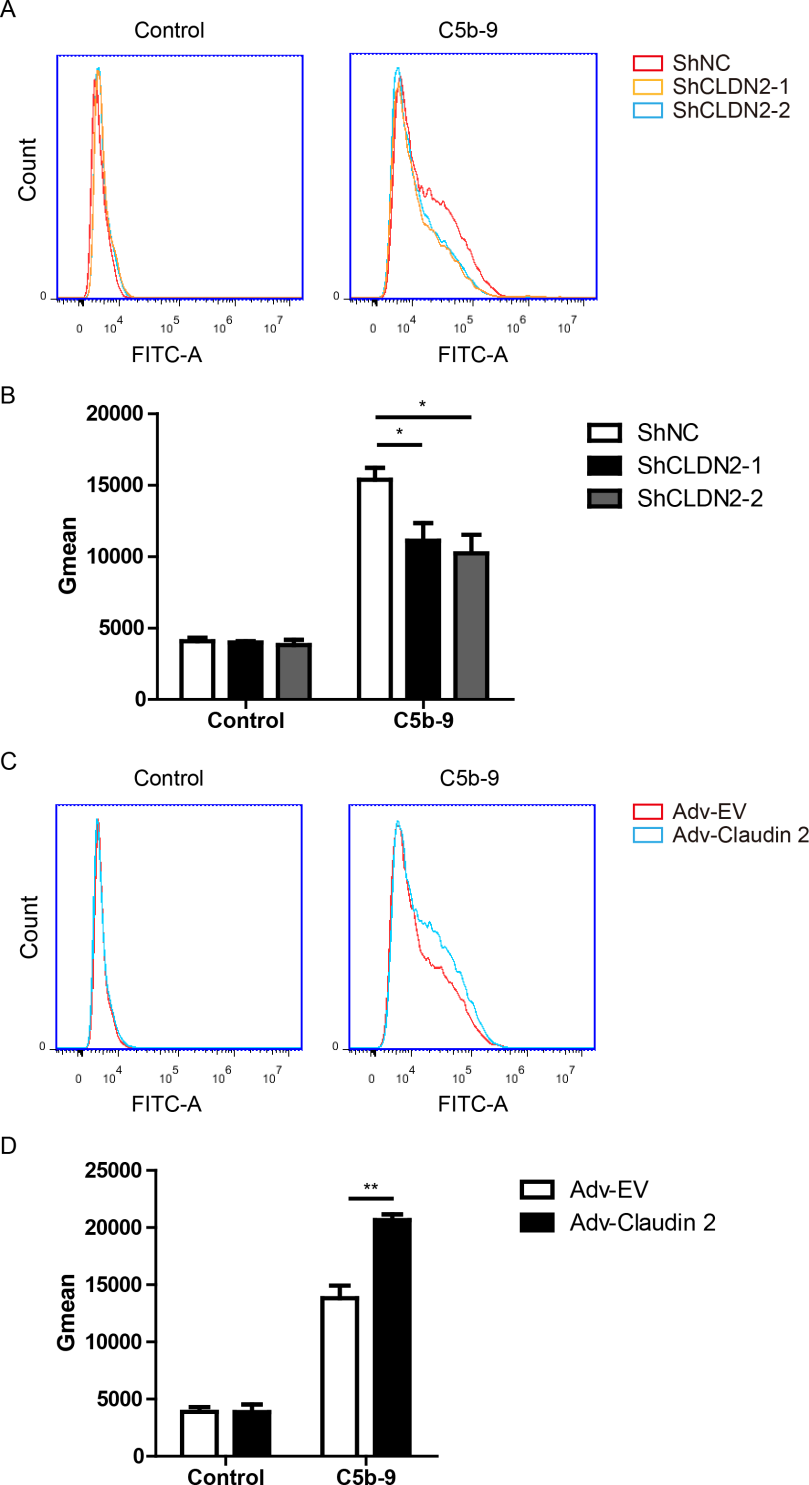
Claudin-2 promotes C5b-9 deposition on PIECs. **(A, B)** PIECs were infected with ShNC, ShCLDN2-1, or ShCLDN2-2 for 4 days, and then incubated with human serum for 30 min. The extent of C5b-9 deposition **(A)** was assessed by flow cytometry. The quantitation data were presented by Gmean **(B)**. **(C, D)** PIECs were infected with Adv-EV or Adv-Claudin-2 for 72h, and then incubated with human serum for 30 min. The extent of C5b-9 deposition **(C)** was assessed by flow cytometry. The quantitation data were presented by Gmean **(D)**. Data are representative of at least three independent experiments (mean ± SEM). *p < 0.05, **p < 0.01 by Student’s t test.

Next, we asked whether Claudin-2 affected complement activation. The C5b-9 complex, also known as the membrane attack complex (MAC), assembles on the surface of target cells, forming pores in the cell membrane that result in cell lysis and death (26). This complex forms during the terminal phase of the complement cascade. We assessed complement activation using C5b-9 deposition using FACS and found that loss of Claudin-2 suppressed C5b-9 deposition in PIECs (**Figures 4A, B**). Adenovirus mediated Claudin-2 overexpression enhanced C5b-9 deposition in PIECs (**Figures 4C, D**). The data suggest that Claudin 2 enhances MAC formation within a human antibody-mediated CDC model.

### Loss of Claudin 2 reduces C3c and C9 deposition on PIECs

**Figure 5:**
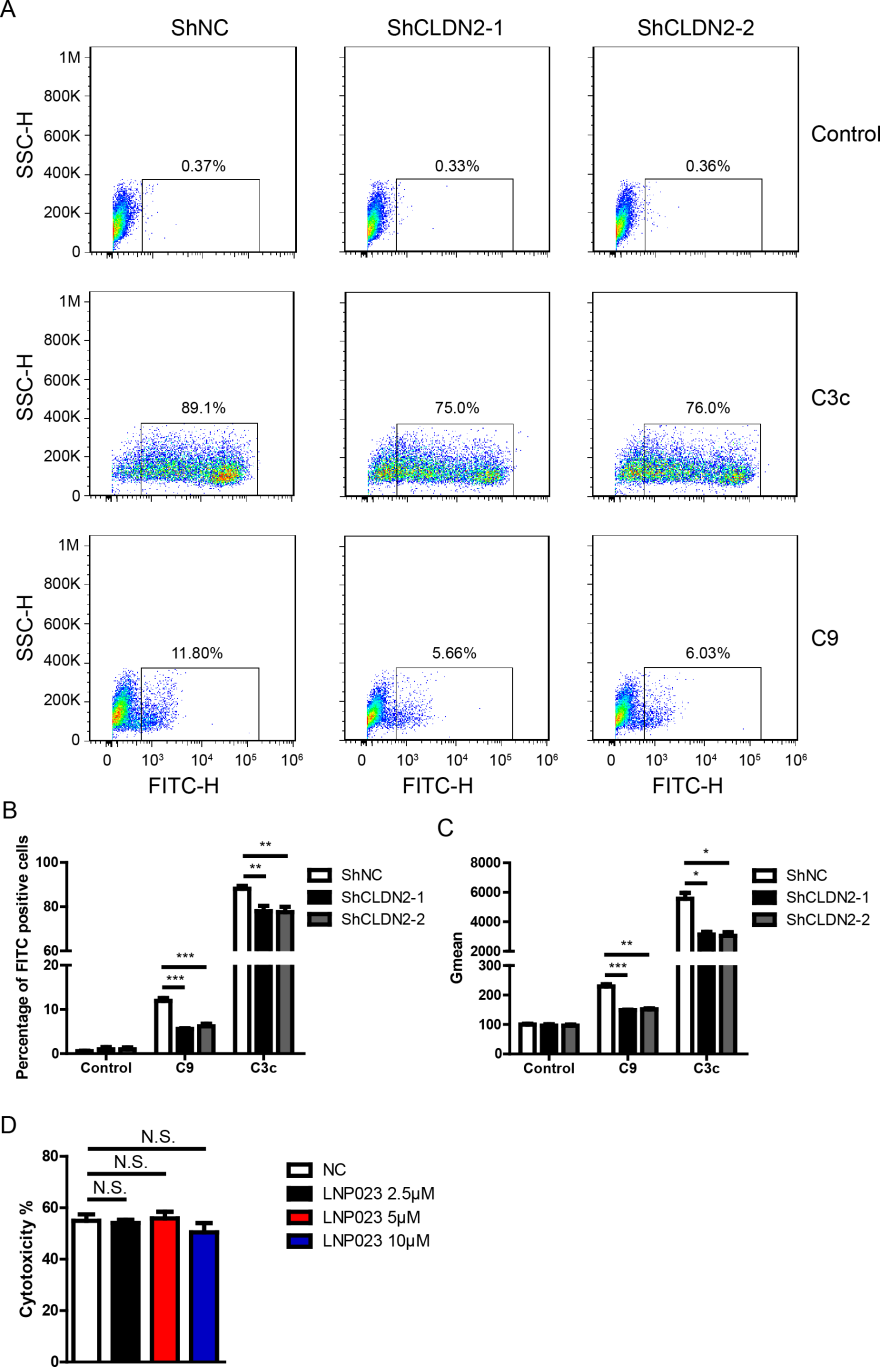
Loss of Claudin 2 decreased C3c and C9 deposition on PIECs. **(A)** PIECs were infected with ShNC, ShCLDN2-1, or ShCLDN2-2 for 4 days, and then incubated with human serum for 30 min. The extent of C3c and C9 deposition was assessed by flow cytometry. **(B)** PIECs were treated as in A, and the percentage of FITC-positive cells was analyzed. **(C)** PIECs were treated as in A, and the quantitation data were presented by Gmean. **(D)** PIECs were pretreated with 2.5 μM, 5 μM, or 10 μM LNP023 or without LNP023 as negative control for 30 min, and then exposed to human serum to induce antibody-mediated CDC. The extent of cytotoxicity was assessed by CCK8. Data are representative of at least three independent experiments (mean ± SEM). *p < 0.05, **p < 0.01, ***p < 0.001 by Student’s t test. N.S. means no significance.

To further confirm the role of Claudin-2 in complement activation, we measured the levels of EC-bound C3 (detected with anti-C3c) and C9 after the PIECs were incubated with human serum. We observed that the number of C3c- and C9-positive cells significantly increased following the incubation of PIECs with human serum (**Figures 5A–C**). Compared to the ShNC group, the ShCLDN2-1 and ShCLDN2-2 groups exhibited fewer C3c- and C9-positive cells (**Figures 5A–C**). Next, we investigated whether the alternative complement pathway was activated in the human antibody-mediated CDC model. PIECs were pretreated with LNP023, an inhibitor of alternative complement pathway targeting factor B, before being exposed to human serum. We found that LNP023 did not affect the cytotoxicity of PIECs in the human antibody-mediated CDC model (**Figure 5D**), suggesting that alternative complement pathway was not activated in the human antibody-mediated CDC model. These data demonstrate that Claudin-2 enhances human antibody-mediated CDC possibly through increasing complement activation and subsequently promoting MAC formation.

### Claudin-2 enhances antibody binding

**Figure 6:**
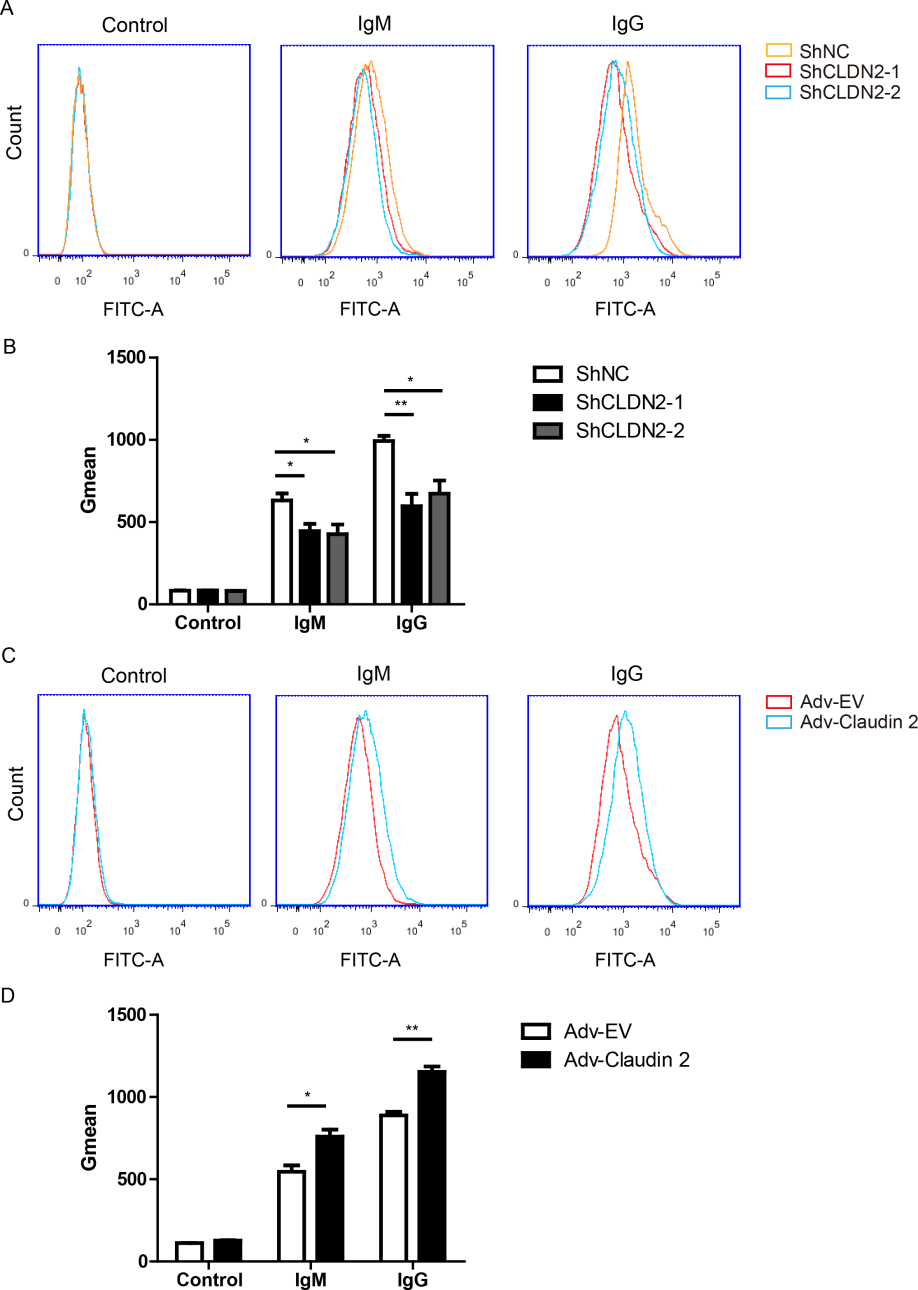
Claudin-2 enhances the extent of human IgG or IgM binding to PIECs. **(A, B)** PIECs were infected with ShNC, ShCLDN2-1, or ShCLDN2-2 for 4 days, and then incubated with heat-inactivated human serum for 30 min. Human IgM and IgG binding to PIECs was measured by flow cytometry **(A)**. The extent of human IgM or IgG binding to PIECs was evaluated by Gmean **(B)**. **(C, D)** PIECs were infected with Adv-EV or Adv-Claudin-2 for 72h, and then incubated with heat-inactivated human serum for 30 min. Human IgM and IgG binding to PIECs was measured by flow cytometry **(C)**. The extent of human IgM or IgG binding to PIECs was evaluated by Gmean **(D)**. Data are representative of at least three independent experiments (mean ± SEM). *p < 0.05, **p < 0.01 by Student’s t test.

Because Claudin-2 is a transmembrane protein (15), so we proposed that Claudin-2 might affect antibodies binding. The binding of IgG or IgM to PIECs significantly increased when the cells exposed to heat-inactivated human serum (HIHS) (**Figure 6A**). Loss of Claudin-2 significantly decrease IgM and IgG binding to PIECs compared to negative control group (**Figures 6A, B**). Overexpression of Claudin-2 in PIECs obviously increased IgG and IgM binding to PIECs (**Figures 6C, D**). Collectively, these data suggest that Claudin-2 enhances human antibody-mediated CDC might through increasing antibodies binding.

### Loss of Claudin-2 enhances cellular permeability but does not modulate cell morphology, proliferation, or apoptosis in PIECs

**Figure 7:**
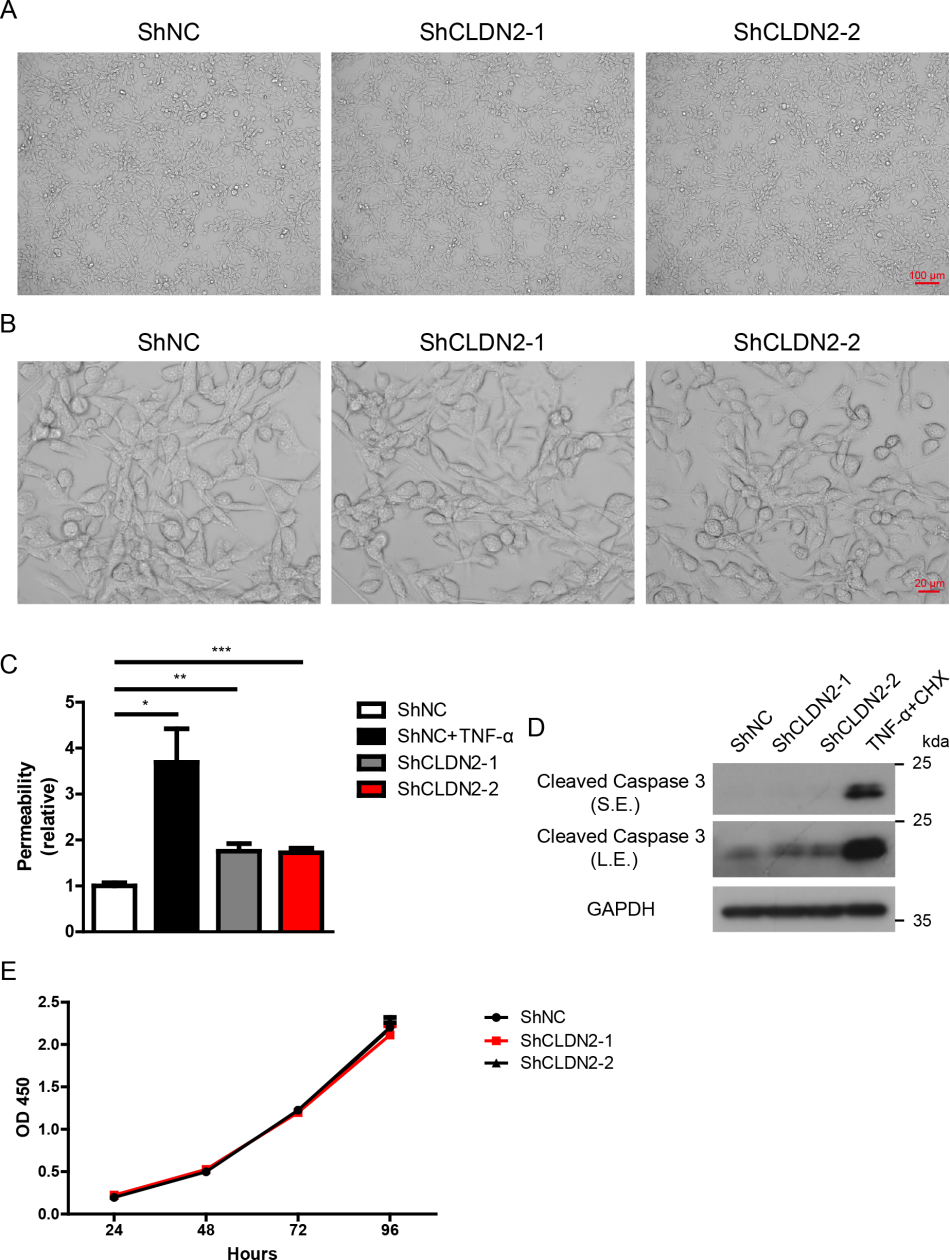
Loss of Claudin 2 increases cellular permeability but does not influence cell morphology, proliferation, or apoptosis in PIECs. **(A, B)** PIECs were infected with ShNC, ShCLDN2-1, or ShCLDN2-2 for 4 days, and their cell morphology was analyzed via microscopy at 100× magnification **(A)** and 400× magnification **(B)**. **(C)** PIECs were infected with ShNC, ShCLDN2-1, or ShCLDN2-2 for 4 days, ShNC-infected PIECs were treated with rhTNF-α (20 ng/ml) for 48 hours as positive control. The permeability of cell monolayer was assessed with trans endothelial flux of FITC-Dextran. **(D)** PIECs were infected with ShNC, ShCLDN2-1, or ShCLDN2-2 for 4 days, ShNC-infected PIECs were treated with recombinant human TNF-α (20 ng/ml) + CHX (10 μg/ml) for 2 hours as positive control. Lysates were analyzed by western blotting with antibodies against Cleaved Caspase 3 and GAPDH. **(E)** PIECs were infected with ShNC, ShCLDN2-1, or ShCLDN2-2 for 4 days, and then their proliferation was assessed with CCK8. Data are representative of at least three independent experiments (mean ± SEM). *p < 0.05, **p < 0.01, ***p < 0.001 by Student’s t test. S.E., short exposure; L.E., long exposure.

Based on the data presented above, we propose that Claudin-2 deficient pigs could potentially enhance the survival of xenografts. Claudin-2 deficient mice exhibited normal appearance, activity, growth, and behavior, but demonstrated altered Na^+^ and water reabsorption in kidney (27, 28). To determine whether the loss of Claudin-2 in pigs results in any adverse effects, we assessed cellular permeability, morphology, proliferation, and apoptosis of PIECs *in vitro*. We observed that the loss of Claudin-2 did not alter cell morphology of PIECs (**Figures 7A, B**). As expected, human recombinant TNF-α significantly increased cellular permeability in PIECs, while the loss of Claudin-2 also caused a slightly increase in cellular permeability (about 1.5-fold) (**Figure 7C**). Moreover, the loss of Claudin-2 did not affect the apoptosis of PIECs, although the combination of TNF-α and cycloheximide (CHX) substantially induced apoptosis of PIECs (**Figure 7D**). We assessed the proliferation of PIECs using CCK8, and found that ShNC, ShCLDN2-1, and ShCLDN2-2 groups exhibited similar proliferative capacity (**Figure 7E**). The data indicate that the loss of Claudin-2 increases cellular permeability but does not regulate cell morphology, proliferation, or apoptosis in PIECs.

## Discussion

Xenotransplantation presents a promising solution to the organ shortage crisis (29, 30). However, a significant limitation of xenotransplantation is immune rejection, as human immune system recognize pig tissues or organs, leading to hyperacute, acute, or chronic rejection (31). Tight junction proteins, such as Claudin-5 and Occludin, have distinct roles in the immune rejection process of xenotransplantation (16, 17). However, the roles of other tight junction proteins in xenotransplantation have been less thoroughly investigated. In this study, we discovered that Claudin-2 significantly enhanced the cytotoxicity of porcine ECs in a human antibody-mediated CDC model. The mechanistic study demonstrated that Claudin-2 increased human antibodies binding to porcine ECs and complement activation, subsequently resulting in injury to the porcine ECs. These findings suggests that Claudin-2 enhances the cytotoxicity of porcine ECs by increasing antibodies binding and complement activation. Our current study uncovers a novel pathological role of Claudin-2 in xenotransplantation.

Previously, we found that tight junction protein Occludin suppressed the cytotoxic effect on porcine ECs in a human antibody-mediated CDC model, whereas the tight junction protein Zo-1 did not affect the cytotoxicity of porcine ECs within the same model (16). A separate study reported that the tight junction protein Claudin-5 protected porcine ECs from CDC mediated by human antibodies (17). In the present study, we found that the tight junction protein Claudin-2 increased the cytotoxicity of porcine ECs in human antibody mediated CDC. Although loss of aforementioned tight junction proteins decreased the barrier function of ECs, these proteins exhibited distinct roles in human antibody mediated CDC model. We proposed that porcine ECs barrier function might not be a significant factor for human antibody mediated CDC.

Complement regulators, such as CD46, CD55, CD59, Factor H, and Factor I, are essential negative regulators that prevent the over-activation of the complement system (12, 25). Previously, we found that TNF-α upregulated the mRNA levels of the complement regulators (CD46, CD55 and CD59), but did not inhibit complement activation (16). In the present study, we found that Claudin-2 did not influence the mRNA levels of complement regulators (CD46, CD55, CD59, Factor H, and Factor I). Notably, the current study revealed that overexpression of Claudin-2 enhanced C3c, C9, and C5b-9 deposition, whereas the loss of Claudin-2 reduced C3c, C9, and C5b-9 deposition, suggesting that Claudin-2 facilitated complement activation and MAC formation. However, the mechanism by which Claudin-2 influences complement activation remains unclear, necessitating further investigation in the near future.

The binding of antibodies is crucial for the cytotoxicity of porcine ECs in the human antibody mediated CDC model. Claudin-2, a transmembrane protein, modulates the binding of IgM and IgG to porcine ECs. Claudin-2 influences antibody binding through several potential mechanisms: (i) Claudin-2 may alter the expression of other antigens, such as the α1,3-galactosyltransferase (GGTA1) and cytidine monophosphate-N-acetylneuraminic acid hydroxylase (CMAH) genes, which are responsible for the synthesis of Galα-1,3-Gal (Gal) and N-glycolylneuraminic acid (Neu5Gc), respectively; (ii) Claudin-2 may serve as a novel xeno-antigen necessary for antibody binding; (iii) As a transmembrane protein, Claudin-2 may directly influence the antibody binding process. To investigate the mechanism by which Claudin-2 regulates antibody binding and subsequently CDC, we plan to explore co-immunoprecipitation (Co-IP) and mass spectrometry (MS) assays to address this question in the near future.

In the present study, we found that the loss of Claudin-2 enhanced cellular permeability but did not affect cell morphology, proliferation, or apoptosis in PIECs. Furthermore, Claudin-2 deficient mice displayed normal appearance, activity, growth, and behavior, however, they showed altered Na^+^ and water reabsorption in kidney (27, 28). Based on these observations, Claudin-2 deficient pigs might exhibited normal appearance, activity, growth, and behavior, however, the function of some organs, such as the kidneys, might decrease.

The limitations of the present study are as follows: (i) The loss of Claudin-2 in porcine ECs reduced human antibody-mediated CDC. However, whether the deficiency of Claudin-2 in pigs decreases immune rejection requires further investigation; (ii) Claudin-2 was found to enhance the cytotoxicity of porcine ECs by modulating antibody binding and complement activation. However, the mechanisms by which Claudin-2 modulates antibody binding and complement activation remain unclear; (iii) It remains unclear whether Claudin-2 increases complement activation by enhancing antibody binding.

In conclusion, in the present study, we found that Claudin-2 enhanced the cytoxicity of porcine ECs in a human antibody mediated CDC. The mechanistic study indicated that antibody binding and complement activation were required for Claudin-2 to increase human antibody mediated CDC. In pig-to-human xenotransplantation, the development of Claudin-2 deficient pig may enhance xenograft survival.

## Data Availability

The raw data supporting the conclusions of this article will be made available by the authors, without undue reservation.
