# Supplementary material for: Claudin-2 enhances human antibody-mediated complement-dependent cytotoxicity of porcine endothelial cells by modulating antibody binding and complement activation
Source: Front Immunol. 2025 Feb 18;16:1547512. doi: 10.3389/fimmu.2025.1547512 (PMC11876394; doi:10.3389/fimmu.2025.1547512)
Supplement: Supplementary file 1 [file Table1.docx]

**Table S1: Listing of primers and primer sequences for real-time PCR**

| **Gene name** | **Primers sequence (5’-3’)** |
| --- | --- |
| Porcine CD46 | acaccaatagccataaggatg |
|  | tccatttgggactactggat |
| Porcine CD55 | gcagactcagtgctctgtct |
|  | ggcactcatattccacggtg |
| Porcine CD59 | ttaggtcacagcctgcagtg |
|  | ggcacggcttcaacgaagat |
| Porcine Claudin 2 | gctggcgaacgagttcttac |
|  | ccttggagaagccgactg |
| Porcine GAPDH | ACAGACAGCCGTGTGTTCC |
|  | ACCTTCACCATCGTGTCTCA |
| Porcine Factor H | GAGTGCATCGAACCTTACGC |
|  | TTGCACTGAGGTGGTTTGGT |
| Porcine Factor I | AGTGTGTCCTTGGATCGTGG |
|  | ATTGGCCTGTGTAAGGCTCC |

**Supplementary Figure 1**


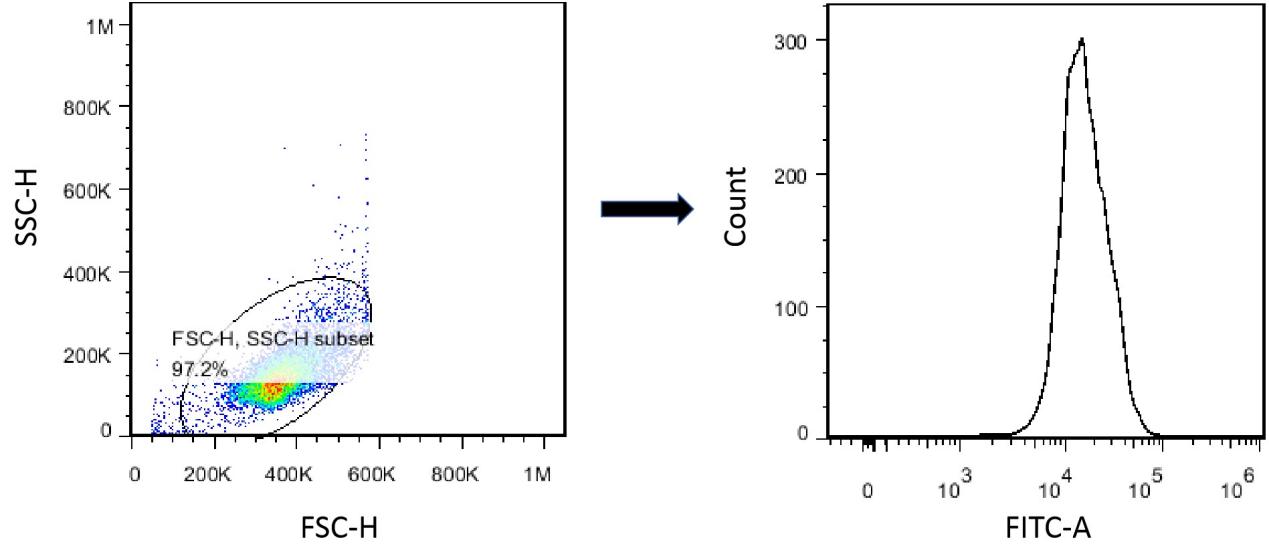


**Figure S1: The gating strategy of CD46.**

**.**

**Supplementary Figure 2**

**
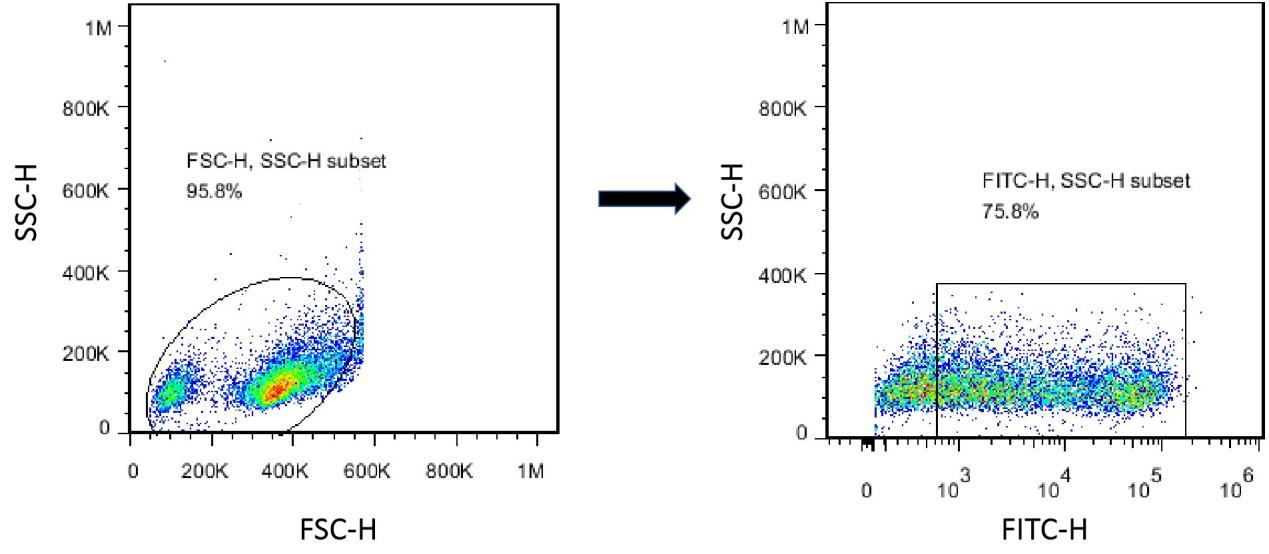
**

**Figure S2: The gating strategy of C9 and C3c.**
